# Oxidative Modification Status of Human Serum Albumin Caused by Chronic Low-Dose Radiation Exposure in Mamuju, Sulawesi, Indonesia

**DOI:** 10.3390/antiox11122384

**Published:** 2022-12-01

**Authors:** Masaru Yamaguchi, Yota Tatara, Eka Djatnika Nugraha, Yuki Tamakuma, Yoshiaki Sato, Tomisato Miura, Masahiro Hosoda, Shinji Yoshinaga, Mukh Syaifudin, Shinji Tokonami, Ikuo Kashiwakura

**Affiliations:** 1 Graduate School of Health Sciences, Hirosaki University, 66-1 Hon-cho, Hirosaki 036-8564, Japan; 2 Graduate School of Medicine, Hirosaki University, 5 Zaifu-cho, Hirosaki 036-8562, Japan; 3 The Research Center for Safety, Metrology, and Nuclear Quality Technology, National Research and Innovation Agency of Indonesia, JI. Lebak Bulus Raya No. 49, Jakarta Selatan 12440, Indonesia; 4 Center for Radiation Research and Education, Nagasaki University, 1-12-4 Sakamoto, Nagasaki 852-8523, Japan; 5 Institute of Radiation Emergency Medicine, Hirosaki University, 66-1 Hon-cho, Hirosaki 036-8564, Japan; 6 Research Institute for Radiation Biology and Medicine, Hiroshima University, 1-2-3 Kasumi, Minami-ku 734-8553, Japan; 7 The Research Center for Radioisotope, Radiopharmaceutical and Biodosimetry Technology, Research Organization for Nuclear Energy, National Research and Innovation Agency, Building 71, Kawasan Puspiptek, Setu, Tangerang Selatan 15310, Indonesia

**Keywords:** high-level natural background radiation area, liquid chromatography-tandem mass spectrometry, oxidative modification profiling, human serum albumin, radiation dose estimation

## Abstract

The recently discovered high-level natural background radiation area (HBRA) of Mamuju in Indonesia provides a unique opportunity to study the biological effects of chronic low-dose radiation exposure on a human population. The mean total effective dose in the HBRA was approximately 69.6 mSv y^−1^ (range: 47.1 to 115.2 mSv y^−1^), based on a re-evaluation of the individual radiation exposure dose; therefore, proteomic analyses of serum components and oxidative modification profiling of residents living in the HBRA were reconducted using liquid chromatography-tandem mass spectrometry. The analysis of the oxidative modification sequences of human serum albumin revealed significant moderate correlations between the radiation dose and the modification of 12 sequences, especially the 111th methionine, 162nd tyrosine, 356th tyrosine, and 470th methionine residues. In addition, a dose-dependent variation in 15 proteins of the serum components was detected in the serum of residents exposed to chronic low-dose radiation. These findings suggest that the alterations in the expression of specific proteins and the oxidative modification responses of serum albumin found in exposed humans may be important indicators for considering the effects of chronic low-dose radiation exposure on living organisms, implying their potential utility as biomarkers of radiation dose estimation.

## 1. Introduction

Human beings are exposed to chronic low-dose radiation through various natural, as well as artificial, radiation sources [1]. It is impossible to completely avoid radiation exposure in our daily lives. External exposure to natural radiation from outer space and the ground, as well as internal exposure to naturally occurring radioactive materials, such as potassium (^40^K), uranium (^238^U), thorium (^232^Th), and their radioactive decay products, e.g., radium (^226^Ra) and radon (^222^Rn) [2], amount to a global effective dose of 2.4 mSv per year (y^−1^). Around the world, there are populations living in high-level natural background radiation areas (HBRA), such as Yangjiang (China), Kerala (India), and Ramsar (Iran), and these residents have much higher natural background radiation exposure (by one to two orders) than the global annual average radiation dose. Particularly in the HBRA of Mamuju, newly discovered by the National Nuclear Energy Agency of Indonesia (BATAN) [3,4,5,6], the potential annual effective doses are even higher than the 20-mSv dose limit for radiation workers, possible because the stable atmospheric conditions suppress the radon diffusion, which leads to increased radon concentrations in the living environment [7].

The linear non-threshold (LNT) hypothesis is the current standard for radiation protection against stochastic effects, such as cancer and hereditary effects. However, insufficient and inconsistent data concerning such low-dose radiation exposure have led to inadequate explanations concerning the LNT, which is the basis for regulatory safety measures. Therefore, the United Nations Scientific Committee on the Effects of Atomic Radiation (UNSCEAR) reported that measurements in HBRAs were important for ensuring the radiation protection of residents and their living environments [8]. Health studies of populations living in HBRAs are a potential source of information on the effects of chronic low-dose/low-dose rate radiation exposure. Thus far, however, there has been no concrete evidence concerning the potential health implications due to exposure <100 mSv y^−1^ [9]. Therefore, it is essential to study the underlying biological mechanisms in response to low-dose/low-dose rate radiation exposure, which may increase our understanding of radiation protection science and its effects on human health. Very recently, we analyzed the serum proteome and oxidative modification profiles of residents of Tande Tande hamlet, as the HBRA (annual effective dose: 49.6 mSv y^−1^), and the Topoyo subdistrict, as a normal-level area (NBRA, 1.22 mSv y^−1^), in Mamuju. Our findings indicated that traces of radiation exposure are recorded in the amino acid sequences of human serum albumin (HSA), and a new methodology may be able to evaluate biological responses around 50 mSv [10,11]. However, since these effective doses were estimated only from the external exposure dose measured by a pocket survey meter, and the internal exposure dose from the breathing of indoor radon, a re-evaluation should be conducted, with more accurate effective doses estimated from all parameters contributing to external and internal radiation exposure caused by radionuclides, especially heavy-metal radionuclides [12].

In the present study, to reassess the health effects among the residents, serum samples were collected from the HBRA residents considered to have a mean total effective dose of 69.6 mSv y^−1^ and the NBRA residents considered to have a mean total effective dose of 4.17 mSv y^−1^ on Mamuju Island. Each sample was subjected to a proteomic analysis and oxidative modification profiling by liquid chromatography-tandem mass spectrometry (LC-MS/MS).

## 2. Materials and Methods

### 2.1. Ethical Statement

The institutional review board statement, approval number, and sate of approval are listed in the “Institutional Review Board Statement” section.

### 2.2. Blood Sampling from the Residents

This study was conducted in Tande Tande hamlet, as the HBRA, and the Topoyo subdistrict, as the NBRA, in coordination with the local health office. Detailed geographic information was provided in previous reports [10]. All volunteers were briefed prior to the study, and each one signed a consent form and completed a questionnaire to provide brief biodata information. Any individuals suffering from an illness or taking medication were excluded. Peripheral blood was collected from the antecubital vein in the morning using BD Vacutainer tubes (Becton Dickinson; Oakville, ON, Canada). The blood was allowed to clot for at least 30 min at room temperature, and then the serum was collected by centrifugation at 1200× *g* for 10 min at 4 °C and stored frozen at −80 °C during transportation from Indonesia to Japan and until the analysis.

### 2.3. External and Internal Radiation Exposure Dose Assessments to Estimate the Total Effective Dose

The effective dose was estimated as the cumulative dose from external and internal exposure. External exposure is due to exposure to environmental gamma radiation, while internal exposure is due to digestion and breathing. To evaluate the personal dose received by the residents, individual measurements of the personal dose equivalent Hp(10) were performed using an optically stimulated luminescence dosimeter (OSLD; Nagase Landauer, Ltd., Ibaraki, Japan), which was calibrated using the ^137^Cs source, with the detector positioned on a phantom. All participants wore the device during the day and placed it near their bedside at the night. Internal exposure through digestion can occur from the ingestion of ^226^Ra, ^232^Th, and ^40^K in the diet and ^222^Rn and ^226^Ra in drinking water, while internal exposure is sustained through breathing by the inhalation of ^222^Rn (radon) and ^220^Rn (thoron). Individual total effective dose calculations were performed based on the methods described in our previous reports [7,12].

### 2.4. LC-MS/MS and High-Resolution Multiple Reaction Monitoring (MRM-HR)

The detailed measurement methods were described in our previous report [10,11]. In brief, serum was diluted with ammonium bicarbonate. The serum proteins were precipitated with acetone and resuspended in ammonium bicarbonate, at which point they were denatured with trifluoroethanol and dithiothreitol (DTT). Free cysteine residues were alkylated with iodoacetamide, which was quenched with DTT. The samples mixed with ammonium bicarbonate were incubated before trypsinization. These peptides were analyzed using a TripleTOF 6600 mass spectrometer (AB Sciex; Framingham, MA, USA). A non-labeled quantitative method (SWATH) was used for the serum proteome analysis. Peptide peak areas were normalized to the sum of the peak areas of all measured peptides. A principal component analysis (PCA) and orthogonal partial least square-discriminant analysis (OPLS-DA) were performed using the Simca software program (Infocom Corp., Tokyo, Japan).

The MRM-HR method was used for the oxidative modification profiling of HSA. An MRM-HR experiment was developed using the Skyline software program (MacCoss Lab, University of Washington, Seattle, WA, USA). The oxidative modification sites of HSA were shown in a previous report [10]. The peak areas obtained were normalized by calculating the relative abundance of each modified peptide using the corresponding non-modified peptide.

### 2.5. Statistical Analyses

We used the Origin 7.5 software program (OriginLab Corp; Northampton, MA, USA) to evaluate the significance of the relationships between the radiation exposure doses and protein expression. We conducted a Spearman rank correlation analysis and calculated the correlation coefficients. The levels of significance were calculated by a Student’s *t*-test or the Mann–Whitney U test using the Excel 2016 software program (Microsoft, Redmond, WA, USA) with the Statcel3 add-on (OMS, Saitama, Japan). *p* values less than 0.05 were considered to indicate statistical significance.

## 3. Results

### 3.1. The Re-Assessment of Each Annual Effective Dose

We re-evaluated the serum samples from residents of Tande Tande hamlet, as an HBRA (13 males and 13 females), and Topoyo district, as an NBRA (14 males and 9 females). The subjects’ ages were similar between the locales (HBRA, median—42 years old; NBRA, median—41 years old). The effective dose was calculated as the sum of the dose received from external exposure and internal exposure. The individual effective dose due to external exposure (external effective dose), individual effective dose due to internal exposure (internal effective dose), and annual total effective dose of the study subjects are summarized in Table 1 (NBRA) and Table 2 (HBRA). The median (range) external effective dose in the HBRA was 9.3 mSv y^−1^ (7.8–11.4 mSv y^−1^). The median NBRA was 0.54 mSv y^−1^ (0.32–0.72 mSv y^−1^). With regard to the internal doses that resulted from inhalation, the median radon concentration in the HBRA was 927 Bq m^−3^ (396–1644 Bq m^−3^), and the median thoron progeny concentration (equilibrium equivalent thoron concentration, EETC) was 22 Bq m^−3^ (13–40 Bq m^−3^). Thus, the median internal effective dose from radon and thoron in the HBRA was 61 mSv y^−1^ (27–108 mSv y^−1^). In the NBRA, the median radon and thoron progeny concentrations were 32 Bq m^−3^ (23–61 Bq m^−3^) and 2.2 Bq m^−3^ (0.9–3.4 Bq m^−3^), respectively, with a median internal effective dose of 3.2 mSv y^−1^ (1.8–5.5 mSv y^−1^). In contrast, the median dose to which an individual is exposed as a result of ingestion of foodstuffs and drinking water was set to approximately 0.30 mSv in the NBRA and approximately 0.52 mSv in the HBRA, according to our previous report [12]. Taken together, the median total effective dose in the NBRA was 4.17 mSv y^−1^ (mean, 4.17 mSv y^−1^; range, 3.30–5.32 mSv y^−1^), while that in the HBRA was 67.8 mSv y^−1^ (mean, 69.6 mSv y^−1^; range, 47.1–115.2 mSv y^−1^). The re-evaluation revealed that the majority of the residents in the HBRA received radiation exposure from natural radionuclides, equivalent to or more than twice the dose limits for occupational exposure to radiation in planned exposure situations (expressed as an effective dose of 20 mSv per year, averaged over a defined 5-year period [100 mSv in 5 years], with the effective dose not exceeding 50 mSv in any single year). It is noted that the estimated dose for all measurements excludes external exposure due to cosmic radiation and internal exposure due to intake of polonium (^210^Po) and plumb (^210^Pb) in foodstuffs and drinking water, which are the radionuclides that account for most of the ingestion dose [7,12,13].

### 3.2. Correlations between Serum Proteomic and Oxidative Modification of HSA Profiling, and Individual Effective Doses of Study Subjects Living in Both Areas

In our previous report regarding a serum proteomic analysis and oxidative modification profiling of HBRA and NBRA residents in Mamuju [10], a proteomic analysis showed that the levels of complement component 6 (C6) and alpha-1-acid glycoprotein 2 (ORM2) of the 19 proteins identified tended to increase with increasing radiation dose exposure. In addition, we focused on the oxidative modification of HSA as a result of chronic low-dose radiation exposure. HSA is a dominant serum protein (approximately 60% of the total proteins) that can function as natural radical scavengers [14,15], and it is known that, as in liver and kidney diseases, along with aging, oxidized forms of albumin accumulate in the blood, making it useful as a biological marker of pathological oxidative stress [15,16]. A total of 38 oxidative modification sites were identified in the amino acid sequence of HSA. Among these, four specific amino acids of HSA, the 162nd and 356th tyrosine residues, and the 111th and 470th methionine residues, showed dose-dependent oxidative modifications.

**Figure 1:**
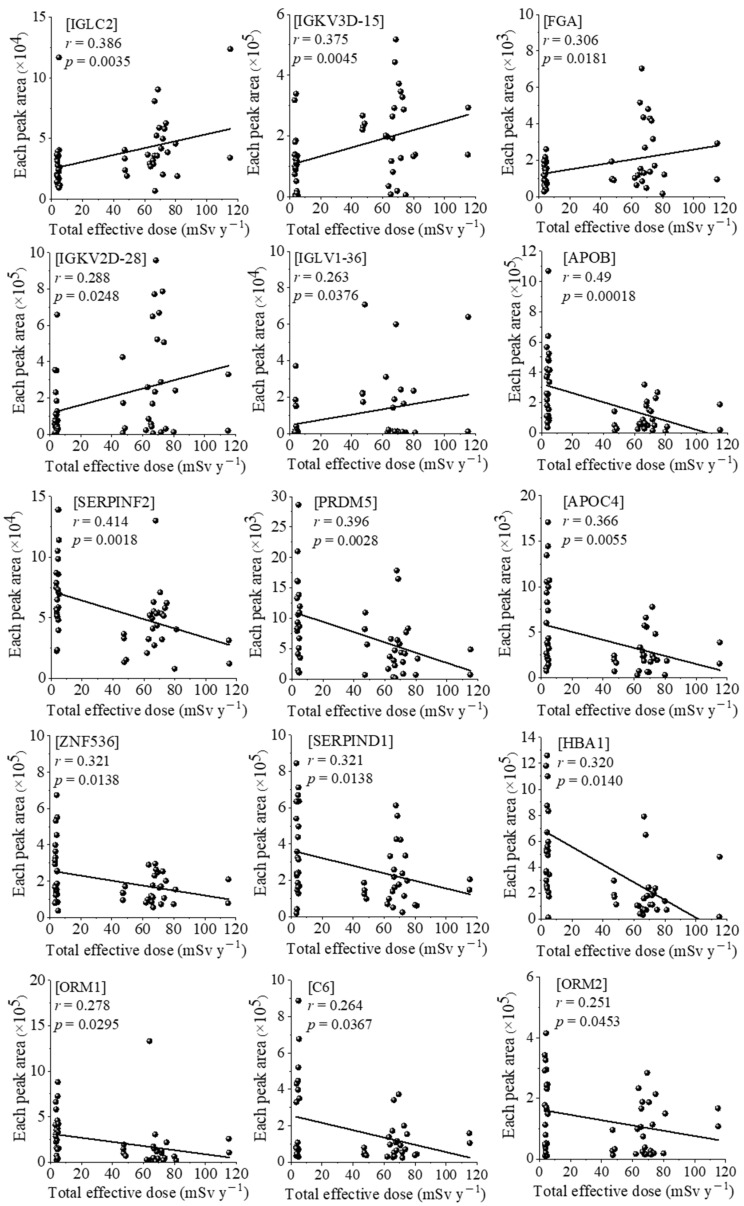
The linear fitting for forecasting the total effective radiation dose using a serum proteome analysis. The linear fitting for forecasting the total effective radiation dose by measuring 15 proteins: IGLC2, IGKV3D−15, FGA, IGKV2D−28, IGLV1−36, APOB, SERPINF2, PRDM5, APOC4, ZNF536, SERPIND1, HBA1, ORM1, C6, and ORM2. R indicates the correlation coefficient value, and *p* < 0.05 was considered statistically significant.

**Figure 2:**
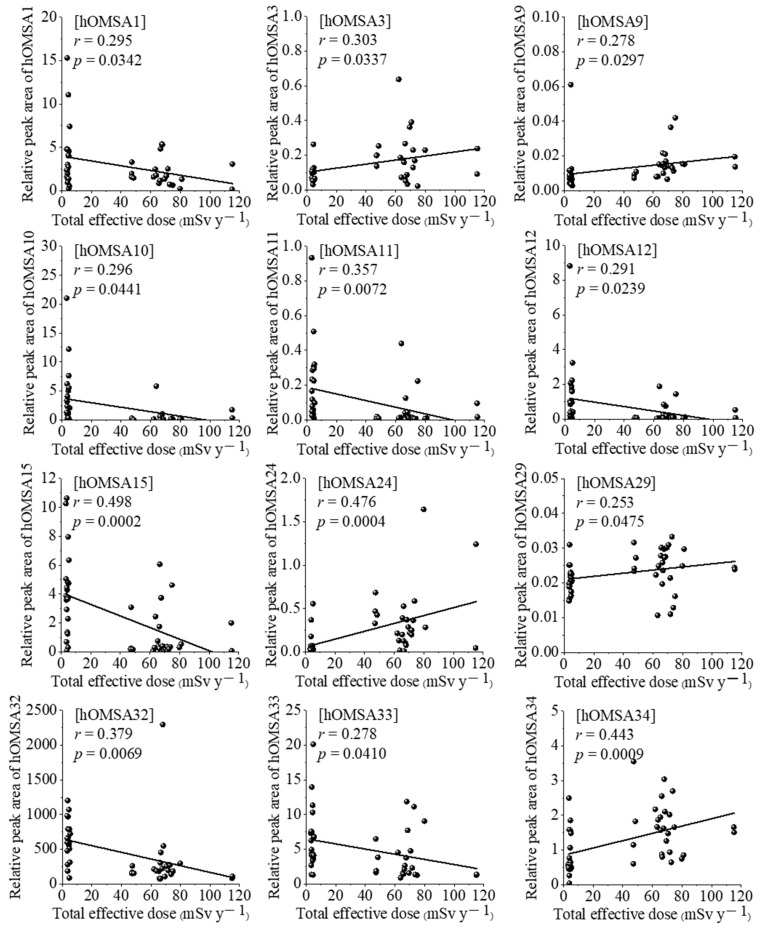
The linear fitting for forecasting the total effective radiation dose using the oxidative modification sequence of HSA. The linear fitting for forecasting the total effective radiation dose by measuring the oxidative modifications of 12 acid amino sequences of HSA: hOMSA1, hOMSA3, hOMSA9, hOMSA10, hOMSA11, hOMSA12, hOMSA15, hOMSA24, hOMSA29, hOMSA32, hOMSA33, and hOMSA34. R indicates the correlation coefficient value, and *p* < 0.05 was considered statistically significant.

Therefore, we determined that the correlations with serum proteomic and oxidative modification of human serum albumin (hOMSA) profiles should be investigated using the re-evaluated annual total effective doses. The correlation between these doses and the expression of 15 proteins is shown in Figure 1. There was a significant positive correlation between the total effective dose and five proteins: immunoglobulin lambda constant 2 (IGLC2), immunoglobulin kappa variable 3D-15 (IGKV3D-15), fibrinogen alpha chain (FGA), immunoglobulin kappa variable 2D-28 (IGKV2D-28), and immunoglobulin lambda variable 1–36 (IGLV1-36) at *r* = 0.386, *r* = 0.375, *r* = 0.306, *r* = 0.288, and *r* = 0.263, with *p* < 0.05, respectively. In contrast, there was a significant negative correlation between the total effective dose and 10 proteins: apolipoprotein B-100 (APOB), alpha-2-antiplasmin (SERPINF2), PR domain zinc finger protein 5 (PRDM5), apolipoprotein C-4 (APOC4), zinc finger protein 536 (ZNF536), heparin cofactor 2 (SERPIND1), hemoglobin subunit alpha 1 (HBA1), ORM1, C6, and ORM2 at *r* = −0.490, *r* = −0.414, *r* = −0.396, *r* = −0.366, *r* = −0.321, *r* = −0.321, *r* = −0.320, *r* = −0.278, *r* = −0.264, and *r* = −0.251, with *p* < 0.05, respectively. The results of the correlation analysis of hOMSA and the total effective doses of NBRA and HBRA residents are shown in Figure 2 and Table 3. There was a significant positive correlation between the total effective dose and 5 types of hOMSAs: hOMSA3 (111th methionine residue, *r* = 0.303, *p* = 0.0337), hOMSA9 (162nd tyrosine, *r* = 0.278, *p* = 0.0297), hOMSA24 (356th tyrosine, *r* = 0.476, *p* = 0.0004), hOMSA29 (419th phenylalanine, *r* = 0.253, *p* = 0.0475) and hOMSA34 (470th methionine, *r* = 0.443, *p* = 0.0009). In contrast, 7 sequences showed a significant negative dose-dependent correlation: hOMSA1 (54th tyrosine, *r* = 0.295, *p* = 0.0342), hOMSA10 (171th proline, *r* = 0.296, *p* = 0.0441), hOMSA11 (174th tyrosine, *r* = 0.357, *p* = 0.0072), hOMSA12 (183th lysine, *r* = 0.291, *p* = 0.0239), hOMSA15 (264th lysine, *r* = 0.498, *p* = 0.0002), hOMSA32 (470th methionine, *r* = 0.379, *p* = 0.0069), and hOMSA33 (476th tyrosine, *r* = 0.278, *p* = 0.0410). Among the identified amino acid sequences of HSA, tyrosine and methionine in particular underwent radiation dose-dependent oxidative modification, a finding that was consistent with our previous report [10].

## 4. Discussion

This study reassessed the health effects among residents of Tande Tande hamlet, as an HBRA (mean total effective dose of 69.6 mSv y^−1^), and Topoyo subdistrict, as an NBRA (mean total effective dose 4.17 mSv y^−1^) (Table 1 and Table 2). The above doses in the HBRA and NBRA were 1.4 and 3.3 times higher, respectively, than those reported in our previous study, since the mean total effective doses in the HBRA and NBRA were 49.6 mSv y^−1^ and 1.26 mSv y^−1^, respectively [10]. The present study reported more comprehensive total effective doses, as it included the dose associated with internal exposure due to the intake of radioactivity in foodstuffs, as well as that due to inhalation of thoron. It should be noted, however, that the present study did not include the contribution of ^210^Po and ^210^Pb in foodstuffs or external exposure due to cosmic radiation. In Mamuju, volcanic rocks containing ^238^U and ^232^Th are the main sources of radiation [17,18], the concentrations of which are 42 and 33 times higher, respectively, than the world average. In addition to the high soil concentrations of ^238^U and ^232^Th, the atmospheric concentrations of radon and thoron are also high. Radon and thoron progeny are the greatest contributors to the total effective dose, accounting for 60% and 25%, respectively, while foodstuffs and drinking water account for 0.77% and 0.01%, respectively. Radon and thoron progeny thus contribute approximately 85% (60 mSv y^−1^) of the total effective dose, while the external effective dose contributes 14% (9.3 mSv y^−1^). With regard to previous studies of HBRAs, a study from Kerala reported that the contribution of external exposure to the total dose was greater than that of internal exposure, while a study from Yangjiang reported the contribution of internal exposure [13]. These differences demonstrate that the dominant pathways of radiation exposure and radiation dose levels differ within each HBRA, and that residents of Mamuju are subject to both internal and external exposure. In Mamuju, the annual effective dose is approximately 30 times higher than the world average. Furthermore, the Geographical area of Mamuju is wide, and the area is heavily populated. Accordingly, Mamuju may be a useful area for conducting risk assessments of HBRAs through epidemiological studies. This may provide a greater understanding of the health effects associated with chronic low-dose radiation exposure.

We detected a significant correlation between the total effective dose and the expression of 15 proteins (Figure 1), suggesting the possibility that these proteins can be used to predict chronic exposure to low-dose radiation. To our knowledge, this is a new finding, as few previous reports have shown an association between low/high-dose ionizing radiation and the expression of these proteins. Among the 5 identified proteins that showed a positive correlation with the radiation dose, FGA, in particular, which is a large, multimeric plasma glycoprotein that is constitutively synthesized by liver hepatocytes, has been reported to function as a biomarker of various diseases via mass spectrometry. Bai et al. speculated the FGA could be a potential biomarker for forecasting relapse, monitoring minimal residual disease, and evaluating therapeutic response in adult acute lymphocytic leukemia patients [19], and several reports have described the involvement of FGA expression in malignant tumors, such as multiple myeloma, hepatocellular carcinoma, colorectal cancer, gastric cancer, and oral squamous cell carcinoma, as well as its role as a poor prognostic factor [20,21,22,23]. Rithidech et al. investigated the radiation-responsive pattern of protein expression profiles in blood plasma using proteomics approaches in mouse models [24]. A total of 18 proteins, including FGA, were significantly upregulated at 2 days post-irradiation, suggesting that alterations in the expression of specific proteins may be indicative of radiation exposure. Conversely, among the 10 identified proteins that showed a negative correlation with the radiation dose, APOB had a particularly high correlation coefficient at *r* = −0.490, with *p* = 0.00018. Low-density lipoproteins, such as APOB, are susceptible to oxidative modification [25], and several reports have shown that a nonnegligible proportion of hydroxy radicals remained able to initiate the oxidation of the APOB protein, leading to APOB carbonylation and fragmentation. This suggests that oxidative stress induced by chronic low-dose radiation exposure may cause dose-dependent fragmentation of the APOB proteins [26,27]. Deng et al. reported that PRDM5 is silenced in human cancers and has developed suppressive activities, and that the inactivation of PRDM5 may play a role in carcinogenesis [28]. In addition, Cheng et al. showed a decrease in ORM1 and an increase in apoptosis upon oxidative stress stimulation in vivo and in vitro [29]. To our knowledge, no studies have investigated the changes in the above-mentioned proteins in association with radiation exposure (especially low-dose exposure). Further validation studies in humans, including the validation of new biomarkers for low-dose radiation, and assessments of the associations with disease and/or carcinogenesis, are therefore necessary.

We also recently developed the MRM-HR method targeting hOMSAs using LS-MS/MS and found several specific sequences (amino acid residues) that undergo dose-dependent oxidative changes [10,11]. Five sequences showed positive dose-dependent correlations, while seven sequences showed negative dose-dependent correlations (Figure 2 and Table 3). Proteins circulating in the blood are known to be a main target of reactive oxygen species (ROS) produced by the interaction of ionizing radiation and water molecules. Proteins undergo oxidative modification through the actions of ROS, which leads to the disruption of their structure and functions. Oxidatively damaged proteins accumulate with aging and due to various pathological conditions [30]. HSA, the most abundant protein in human blood, has a relatively long half-life in blood (approximately three weeks) in comparison to other serum proteins. Since it is consequently exposed to various active chemical species at high frequencies, HSA undergoes post-translational modifications, such as oxidation, glycosylation, dimerization, and carbamylation; thus, its analysis can provide information on oxidative stress in the systemic circulation [31]. Increased oxidized serum albumin levels have been reported in patients with hypertension, diabetes mellitus, liver disease, and renal failure, as well as in individuals who have performed strenuous exercise and the elderly population [32]. These findings were previously considered to have occurred solely due to oxidative stress. However, a number of recently published ex vivo studies have reported that these pathological conditions can be aggravated by oxidized serum albumin. In addition, the serum albumin redox state has recently been demonstrated to be a novel biomarker that reflects the protein nutritional status with high sensitivity [33,34]. Among the identified amino acid sequences, tyrosine and methionine, in particular, underwent radiation dose-dependent oxidative modification in response to increased levels of intracellular oxidative stress, and these results were consistent with those from our previous report, where oxidative modifications of the 111th methionine, 162nd tyrosine, 356th tyrosine, and 470th methionine were dose-dependent, even after re-evaluating the radiation exposure dose [10]. HSA consists of three folded homologous regions (domains), and residues 1 to 195 correspond to domain I. Interestingly, half of the identified HSA oxidation-modified sequences are located in domain I, and overall, the N-terminal domain I of HSA is associated with chronic low-dose radiation exposure. Momozono et al. showed that the oxidation of the 147th methionine residue of HSA was significantly higher in diabetic patients than in healthy subjects [35,36]. Although there are differences between the free radicals generated by oxidative stress and those generated by radiation exposure, of note, the domains of albumin that are sensitive to oxidation reactions are consistent between the two situations. Several epidemiological studies in HBRAs around the world (Ramsar, Yangjiang, Kerala, and Guarapari) have reported no correlation between radiation exposure and cancer rate or mortality [37,38,39,40]. Furthermore, no reports have described the biological components at the molecular level. This study reconfirms the possibility that the changes in the expression of specific proteins and the oxidative modification responses of serum albumin found in exposed humans are important indicators for considering the effects of chronic low-dose radiation exposure on living organisms, suggesting their possible application as biomarkers of radiation dose estimation in the future. However, the findings of the present study should be interpreted with caution, as the study population was relatively small due to various limitations regarding the research. Therefore, the functional changes of oxidatively modified albumin, the subsequent effects on individual health, disease, and longevity, as well as the relationship with radiation damage should be investigated through a follow-up study in the HBRA and a validation analysis in human low-dose exposure cases in future studies.

Our previous report showed that the mean (range) lifetime cumulative dose of the residents of Mamuju was 2.2 Sv (1.2–8.1 Sv), which is much higher than that of atomic bomb survivors (200 mSv), as well as for most other exposed populations [12]. Studies on atomic bomb survivors have shown that radiation disrupts the homeostasis of T cells in the body, causing persistent inflammation through increased production of inflammatory cytokines, such as tumor necrosis factor α and interleukin-6, and inducing cardiovascular and other diseases [41]. Public health data from the Mamuju residents indicate that the main diseases are upper respiratory tract infections, gastritis, and hypertension, with symptoms differing among individuals [12]. In the LC-MS/MS analysis, no significant elevation of inflammatory cytokines was observed (data not shown), and at present, there are no reports showing a strong association between identified protein or oxidative modification of albumin and these diseases, as revealed in the present study. However, the situation should be further investigated epidemiologically. In addition, we also found that most houses in the HBRA had ^222^Rn activity concentrations exceeding the reference level of the World Health Organization, and 40% of the houses exceeded the International Commission on Radiological Protection (ICRP) recommendations [12]. Radon exposure is, after smoking, the second-most common cause of lung cancer (between 3% and 14% of cases) [42]. About half of Mamuju residents are smokers, but there is no information on the precise incidence of lung cancer, as no detailed epidemiological studies have been conducted in this population. However, the absolute risk of lung cancer caused by radon exposure is significantly higher for smokers than for nonsmokers [43]. Furthermore, regarding the induction of non-cancer diseases, the ICRP states, “the commission judges that the data available do not allow for their inclusion in the estimation of detriment following low radiation doses, less than about 100 mSv” [44]. The fact that Mamuju residents continue to chronically undergo oxidative modification of their serum albumin is certainly a subject worthy of continuous observation. Since the effect of low dose-rates on health and cancer risks after exposure to radiation remains unclear at present, based on this situation, Mamuju is an area ripe for the epidemiological study of health risks (relative to both cancer and non-cancer) due to the chronic low-dose rate radiation emitted from environmental sources.

## Figures and Tables

**Table 1:** Estimated annual effective dose of study subjects living in NBRA.

No	Sex	Age (Years)	External Dose	Internal Dose-Inhalation-		Internal Dose-Ingestion-			Total Dose Effective Dose (mSv)
			OSLD (mSv)	Radon Dose (mSv)	Thoron Dose (mSv)	Radon Water (mSv)	Radium Water (mSv)	Food (mSv)	
1	M	43	0.58	1.5	1.2	0.006	0.003	0.3	3.6
2	F	41	0.61	1.5	1.2	0.006	0.003	0.3	3.6
3	M	70	0.51	1.3	2.1	0.006	0.003	0.3	4.2
4	M	51	0.50	2.1	1.7	0.006	0.003	0.3	4.7
5	M	27	0.32	1.5	2.0	0.006	0.003	0.3	4.2
6	F	49	0.60	2.9	0.7	0.006	0.003	0.3	4.4
7	M	32	0.70	1.2	1.6	0.006	0.003	0.3	3.8
8	M	33	0.68	1.2	2.5	0.006	0.003	0.3	4.8
9	M	50	0.57	1.8	0.7	0.006	0.003	0.3	3.3
10	F	30	0.72	1.5	2.5	0.006	0.003	0.3	5.1
11	M	48	0.53	1.1	1.5	0.006	0.003	0.3	3.4
12	F	40	0.53	2.1	1.7	0.006	0.003	0.3	4.7
13	F	34	0.49	1.5	1.6	0.006	0.003	0.3	3.9
14	M	42	0.54	2.9	1.6	0.006	0.003	0.3	5.3
15	M	41	0.56	2.1	1.7	0.006	0.003	0.3	4.7
16	F	25	0.56	2.1	1.7	0.006	0.003	0.3	4.7
17	M	43	0.42	1.7	1.6	0.006	0.003	0.3	4.0
18	M	37	0.47	1.5	1.6	0.006	0.003	0.3	3.9
19	M	42	0.66	1.7	1.8	0.006	0.003	0.3	4.4
20	F	42	0.70	1.7	1.8	0.006	0.003	0.3	4.5
21	F	45	0.53	1.1	1.5	0.006	0.003	0.3	3.4
22	M	54	0.44	1.6	1.0	0.006	0.003	0.3	3.4
23	F	20	0.39	1.1	1.9	0.006	0.003	0.3	3.7

**Table 2:** Estimated annual effective dose of study subjects living in HBRA.

No	Sex	Age (Years)	External Dose	Internal Dose-Inhalation-		Internal Dose-Ingestion-			Total Dose Effective Dose (mSv)
			OSLD (mSv)	Radon Dose (mSv)	Thoron Dose (mSv)	Radon Water (mSv)	Radium Water (mSv)	Food (mSv)	
1	M	38	7.8	78.2544	28.462	0.00228	0.00222	0.5132	115
2	F	29	8.0	78.2544	28.462	0.00228	0.00222	0.5132	115
3	M	37	9.1	44.1252	15.729	0.00222	0.00229	0.5130	69
4	M	55	10.1	26.0848	10.486	0.00222	0.00229	0.5130	47
5	F	42	10.2	44.1252	15.729	0.00222	0.00229	0.5130	71
6	F	41	7.8	44.3156	14.98	0.00222	0.00228	0.5130	68
7	F	28	11.3	44.1252	15.729	0.00222	0.00228	0.5130	72
8	M	74	8.6	44.3156	11.984	0.00228	0.00229	0.5132	65
9	M	74	7.8	36.414	20.972	0.00228	0.00229	0.5130	66
10	F	63	8.5	36.414	20.972	0.00228	0.00229	0.5128	66
11	M	75	9.4	26.9892	10.486	0.00228	0.00229	0.5130	47
12	F	49	10.7	33.7008	29.96	0.00220	0.00229	0.5132	75
13	M	29	9.5	33.7008	29.96	0.00220	0.00228	0.5128	74
14	F	26	10.5	46.886	23.219	0.00228	0.00229	0.5130	81
15	F	27	9.9	42.9352	9.737	0.00220	0.00229	0.5132	63
16	M	33	8.8	42.9352	9.737	0.00228	0.00229	0.5130	62
17	M	73	8.7	45.3152	17.227	0.00228	0.00229	0.5130	72
18	F	59	8.8	17.5644	20.223	0.00216	0.00229	0.5130	47
19	F	63	9.8	45.3152	17.227	0.00228	0.00229	0.5128	73
20	M	52	8.7	44.3156	14.98	0.00220	0.00229	0.5130	69
21	M	31	9.6	44.3156	11.984	0.00228	0.00228	0.5130	66
22	M	29	9.3	46.886	23.219	0.00228	0.00229	0.5130	80
23	F	59	10.5	26.9892	10.486	0.00216	0.00229	0.5132	49
24	F	26	7.8	44.3156	11.235	0.00228	0.00229	0.5130	64
25	F	44	11.4	38.8416	17.227	0.00228	0.00228	0.5128	68
26	M	20	10.1	38.8416	17.227	0.00216	0.00229	0.5128	67

**Table 3:** Oxidative modification pattern of peptide sequence constituting human serum albumin.

hOMSA ID	Peptide Sequence and Modification Site	Amino Acid Residue	Modification Type
1	DLGEENFKALVLIAFAQY[Oxi]LQQC[CAM]PFEDHVK	54th tyrosine	Oxidation
3	SLHTLFGDKLC[CAM]TVATLRETYGEM[Oxi]ADC[CAM]C[CAM]AK	111th methionine	Oxidation
9	Y[Oxi]LYEIAR	162nd tyrosine	Oxidation
10	RHP[Oxi]YFYAPELLFFAK	171th proline	Oxidation
11	RHPYFY[Oxi]APELLFFAK	174th tyrosine	Oxidation
12	RHPYFYAPELLFFAK[LAA]	183th lysine	Lys -> Allysine
15	LVTDLTK[LAA]VHTEC[CAM]C[CAM]HGDLLEC[CAM]ADDRADLAK	264th lysine	Lys -> Allysine
24	DVFLGMFLY[Oxi]EYAR	356th tyrosine	Oxidation
29	QNC[CAM]ELF[Oxi]EQLGEYK	419th phenylalanine	Oxidation
32	RM[Oxi]PC[CAM]AEDYLSVVLNQLC[CAM]VLHEK	470th methionine	Oxidation
33	RM[Oxi]PC[CAM]AEDY[Oxi]LSVVLNQLC[CAM]VLHEK	476th tyrosine	Oxidation
34	M[Oxi]PC[CAM]AEDYLSVVLNQLC[CAM]VLHEK	470th methionine	Oxidation

## Data Availability

The proteomic data are also available online using accession numbers “PXD025946, PXD025947, PXD025950, and PXD025949” for Proteome Xchange [45] and accession numbers “JPST001164, JPST001165, JPST001168, and JPST001167” for jPOST Repository [46]. Any additional data supporting the findings of this study are available from the corresponding author upon reasonable request.
